# Deterministic Tumor Evolution

**DOI:** 10.1371/journal.pbio.0020284

**Published:** 2004-08-17

The essential difference between cancer cells and normal cells is that cancer cells evolve. Most cancers arise from a single cell through a sequential evolutionary process of mutation and selection. Cancer cells harbor mutations in a number of critical genes that, at various stages during the evolution of the tumor, provide those cells with a selective advantage. Many of the phenotypes, or physical outcomes, conferred by these mutant genes are subverted from a normal cell's repertoire, including proliferation, invasion, migration, loss of differentiation, and loss of apoptosis (programmed cell death); other phenotypes, such as immortalization, are novel. Tumor evolution is thought to adhere to Darwinian principles, with mutations arising randomly within an individual cell, followed by selection for mutant clones with favorable traits.

Support for this idea stems from the observation that end-stage tumors have mutations in a number of genes. But linking mutations in particular genes with defined stages is difficult for most human cancers and especially so for the last and deadliest stage: when cancer disseminates throughout the body during metastasis. It's unclear, for example, whether there are mutations in particular genes or sets of genes that enhance metastasis. That is, do metastatic lesions develop through a continuation of Darwinian evolution, or is metastasis an intrinsic property of the primary tumor, meaning that further genetic evolution is not required? It is also unclear whether there is a “preferred” sequence of mutations, such that selective pressure for particular mutations depends on preexisting mutations.[Fig pbio-0020284-g001]

**Figure pbio-0020284-g001:**
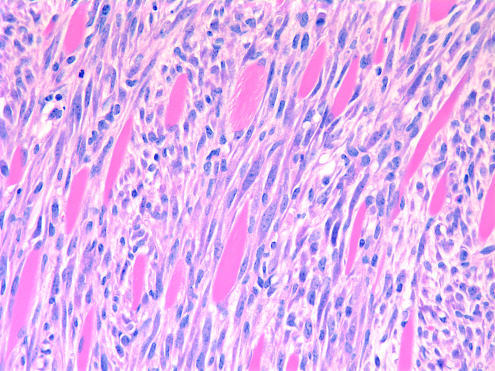
Squamous cell carcinoma invading muscle layer from *ARF*-null mouse

Since the earliest days of research on oncogenes—genes that can cause a cell to become cancerous—it has been known that certain oncogenic mutations cooperate to transform normal cells into cancer cells. For example, an “activating” mutation in the oncogene *Ras* and the loss of the tumor suppressor *p53* cooperate to transform cells.

The paper by Christopher Kemp and his colleagues at the Fred Hutchinson Cancer Research Center sheds light on some of these questions, and many of the issues center around the most notorious oncogene: *Ras*. Using a well characterized mouse model of squamous cell carcinogenesis, which generates a form of skin cancer, the authors examine both the functional and evolutionary relationships between three cancer genes that play major roles in most human cancers: *Ras* and the tumor suppressors *Arf* and *p53*.

Two seminal early observations set the stage: mutational activation of *Ras* is the initiating genetic event in this cancer model, while mutation of *p53* occurs later, during the benign to malignant transition; and expression of mutant *Ras* in cells activates *p53* via signaling through the protein encoded by *Arf*.

Kemp et al. confirm that this pathway is active in “autochthonous” tumors—which grow and develop where they are initiated—by showing that *p53* expression in tumors with *Ras* mutations is dependent on the presence of *Arf*. Thus, during the early benign stages of tumor growth, *Ras* activates *Arf*, which in turn activates *p53*, thereby inhibiting tumor progression. This provides strong selective pressure in favor of cells with mutations in either *Arf* or *p53*, and these mutations are indeed observed as the tumors progress to malignancy. That *Arf* and *p53* function as tumor suppressors was confirmed by demonstrated accelerated tumor progression in mice lacking either *Arf* or *p53*. This answers a longstanding question concerning the nature of the signal that activates *p53* during autochthonous tumor development: Mutation of *Ras* not only initiates tumor development but, through its intracellular signaling through *Arf* and *p53*, directly influences the subsequent evolutionary trajectory of the tumors. In this view, secondary evolutionary events are determined by the preexisting genetic lesion, as a result of direct signaling interactions.

The authors go on to show that tumors lacking *Arf* or *p53* show accelerated metastatic dissemination, a phenomenon rarely seen in mouse squamous cell cancer models. Thus both benign and malignant tumors lacking these tumor suppressors are at high risk for metastasis. As *Ras* is well known to confer many phenotypes required for the metastatic process, it appears that *Ras*, together with loss of its inhibitors, *Arf* and *p53*, may be sufficient to drive this process. More direct evidence that metastasis does or does not require further genetic evolution awaits.

